# Evaluation of Bayesian spatiotemporal infectious disease models for prospective surveillance analysis

**DOI:** 10.1186/s12874-023-01987-5

**Published:** 2023-07-22

**Authors:** Joanne Kim, Andrew B. Lawson, Brian Neelon, Jeffrey E. Korte, Jan M. Eberth, Gerardo Chowell

**Affiliations:** 1 Department of Biomedical Informatics, The Ohio State University, Columbus, OH USA; 2 Department of Public Health Sciences, Medical University of South Carolina, Charleston, SC USA; 3 Usher Institute, Centre for Population Health Sciences, Edinburgh Medical School, University of Edinburgh, Edinburgh, UK; 4 Department of Health Management and Policy, Drexel University, Philadelphia, PA USA; 5 Department of Population Health Sciences, Georgia State University, Atlanta, GA USA

**Keywords:** Bayesian spatiotemporal analysis, Infectious disease outbreak modeling, Public health surveillance

## Abstract

**Background:**

COVID-19 brought enormous challenges to public health surveillance and underscored the importance of developing and maintaining robust systems for accurate surveillance. As public health data collection efforts expand, there is a critical need for infectious disease modeling researchers to continue to develop prospective surveillance metrics and statistical models to accommodate the modeling of large disease counts and variability. This paper evaluated different likelihoods for the disease count model and various spatiotemporal mean models for prospective surveillance.

**Methods:**

We evaluated Bayesian spatiotemporal models, which are the foundation for model-based infectious disease surveillance metrics. Bayesian spatiotemporal mean models based on the Poisson and the negative binomial likelihoods were evaluated with the different lengths of past data usage. We compared their goodness of fit and short-term prediction performance with both simulated epidemic data and real data from the COVID-19 pandemic.

**Results:**

The simulation results show that the negative binomial likelihood-based models show better goodness of fit results than Poisson likelihood-based models as deemed by smaller deviance information criteria (DIC) values. However, Poisson models yield smaller mean square error (MSE) and mean absolute one-step prediction error (MAOSPE) results when we use a shorter length of the past data such as 7 and 3 time periods. Real COVID-19 data analysis of New Jersey and South Carolina shows similar results for the goodness of fit and short-term prediction results. Negative binomial-based mean models showed better performance when we used the past data of 52 time periods. Poisson-based mean models showed comparable goodness of fit performance and smaller MSE and MAOSPE results when we used the past data of 7 and 3 time periods.

**Conclusion:**

We evaluate these models and provide future infectious disease outbreak modeling guidelines for Bayesian spatiotemporal analysis. Our choice of the likelihood and spatiotemporal mean models was influenced by both historical data length and variability. With a longer length of past data usage and more over-dispersed data, the negative binomial likelihood shows a better model fit than the Poisson likelihood. However, as we use a shorter length of the past data for our surveillance analysis, the difference between the Poisson and the negative binomial models becomes smaller. In this case, the Poisson likelihood shows robust posterior mean estimate and short-term prediction results.

**Supplementary Information:**

The online version contains supplementary material available at 10.1186/s12874-023-01987-5.

## Background

The COVID-19 pandemic for the last 3 years has highlighted the importance of public health surveillance. Pandemic data collection efforts became the center stage for the state-of-the-art technology and resources of government, research institutions, and technology firms. Each state health authority, research institutions, and technology firms constructed COVID-19 dashboards to track and guide interventions. For example, South Carolina constructed the COVID-19 dashboard, and technology companies such as Google and Apple provided related data such as mobility and community spread reports in the public domain [1–3]. With these efforts, public health authorities can collect extensive COVID-19 data and make faster inferences and predictions about the current outbreak trend compared to previous infectious disease surveillance activities. The public health surveillance process was defined as “*[T]he ongoing, systematic collection, analysis and interpretation of health data essential to the planning, implementation, and evaluation of public health practice, closely integrated with the timely dissemination of these data to those who need to know*” [4]. After the collection of the data, public health surveillance data analysis can be implemented in two ways regarding its purpose: prospective surveillance analysis and retrospective analysis [5]. Retrospective analysis investigates past trends and aims to find previous change points and important factors affecting disease spread. In contrast, prospective surveillance analysis is about real-time inference for outbreak detection and future prediction based on currently available data. For accurate prospective surveillance, Bayesian spatiotemporal statistical models have been utilized to develop the surveillance metric and the method to deal with reporting delay and under-reporting problems. For example, Corberán-Vallet and Lawson [6] presented a novel surveillance metric to detect the beginning of an infectious disease outbreak. Surveillance Conditional Predictive Ordinate (SCPO) compares the current and estimated disease cases using Bayesian conditional predictive ordinate and identifies the emergence of the disease clusters. Rotejanaprasert et al. [7] and McGaugh [8] developed the nowcasting method to accurately estimate the complete disease case distribution using both Poisson and the negative binomial likelihoods.

So far, Bayesian spatiotemporal surveillance models for the prospective surveillance analysis have mainly used the Poisson likelihood to model various infectious disease outbreaks count data [6, 9, 10]. However, the COVID-19 pandemic challenges us to develop a more flexible modeling framework to accommodate large disease counts and fluctuations. When disease cases are increasing sharply in a short period (e.g., first wave of COVID-19 in the New England regions in 2020 and Omicron waves in the US in winter 2021–2022), the negative binomial likelihood is more flexible to accommodate this over-dispersed infectious outbreak data with its additional dispersion parameter.

The negative binomial likelihood has been used to model over-dispersed count data in many application areas, such as genetics and traffic accident literature [11–13]. It has also been used for infectious disease surveillance [8, 14, 15] but not used widely in a Bayesian spatiotemporal analysis with the prospective surveillance setting. Several nowcasting papers used negative binomial likelihood for Bayesian spatiotemporal modeling to deal with the over-dispersed data [8, 16].

Define ${y}_{ij}$ as the disease count at area i and time j, where i = 1…M, j = 1…T. We denote ${{Y}}_{{ij}}\sim Poisson \left( {\mu }_{ij}\right)$ if

$$P\left({Y}_{ij}={{y}_{ij}}| {\mu }_{ij}\right)=\frac{{e}^{-{\mu }_{ij}}\cdot {\mu }_{ij}^{{y}_{ij}}}{{y}_{ij}!} \,where\, {{E}({Y}_{ij}})=Var\left({Y}_{ij}\right)= {\mu }_{ij}$$

Note that there are multiple negative binomial parameterizations. The negative binomial model parameterization with mean and dispersion ( ${\mu }_{ij}, {r}_{ij}$) is used in this paper. We are interested in the behavior of ${\mu }_{ij}\, and\, {r}_{ij}$ depending on the change in infectious disease counts ${y}_{ij},$ so this parameterization is suitable for our purpose. We denote ${{Y}}_{{ij}}\sim NB \left( {\mu }_{ij}, {r}_{ij}\right)$ if

$$\begin{array}{c}P\left({Y}_{ij}={{y}_{ij}}| {\mu }_{ij}, {r}_{ij}\right)=\left(\frac{{y}_{ij}+{r}_{ij}-1}{{y}_{ij}}\right)\cdot {\left(\frac{{r}_{ij}}{{\mu }_{ij}+{r}_{ij}}\right)}^{{r}_{ij}}\cdot {\left(\frac{{\mu }_{ij}}{{\mu }_{ij}+{r}_{ij}}\right)}^{{y}_{ij}}\\ where\, {{E}({Y}}_{{ij}})={\mu }_{ij}, Var\left({Y}_{ij}\right)=\frac{{\mu }_{ij}^{2}}{{r}_{ij}}+{\mu }_{ij}\end{array}$$

In this study, we investigate the logarithm of the mean ${\mu }_{ij}$ for the Poisson and the negative binomial likelihoods with various spatial terms and epidemic effect terms to model the infectious disease outbreak.

Prospective surveillance requires us to accommodate on-demand analysis and frequent changes in outbreak trends, so we need to consider how much past data we use for our modeling of outbreak trends. In the time-series forecasting analysis, the length of the past data usage is called the ‘window size’, and it is an important consideration for the forecasting process when the disease trend has a large variation [17, 18]. When the outbreak trend is constantly changing, using a longer window size might be irrelevant and add more noise with out-of-date information. However, including too short time windows can give insufficient information to the model and increase the instability of the model fit. From the forecasting literature, Bayesian spatiotemporal surveillance has adopted a “sliding window” approach for prospective surveillance analysis [6, 7]. As time passes, it uses a fixed number of previous time periods for the analysis by dropping the oldest time periods from the dataset for analysis. This approach gives the computational efficiency for the Markov Chain Monte Carlo (MCMC) computation and enables the surveillance model to adapt to a fast-changing environment. For example, Corberán-Vallet and Lawson [6] used a sliding window approach to analyze the early detection metric. They compared the metric’s performance depending on the different window sizes in the weekly salmonellosis outbreak data. Rotejanaprasert et al. [7] implemented real-time surveillance with the system to estimate the reporting delays. The Poisson and the negative binomial likelihoods with spatiotemporal mean models have been used for infectious disease surveillance studies. It was assumed that the negative binomial likelihood was used for over-dispersed data.In contrast, the Poisson likelihoods were used for those without an over-dispersed pattern. However, the performance of mean models and likelihoods can be changed depending on how much past data was used. Therefore, we would like to comprehensively evaluate the Poisson and the negative binomial data models with various infectious disease mean models with various window sizes. The novelty of our paper lies in evaluating both Poisson and the negative binomial likelihoods, along with various Bayesian spatiotemporal mean models, while different lengths of the past data for the prospective surveillance were analyzed.

Specifically, we would like to answer the following questions:

1. Comparison between likelihoods: When we have the over-dispersed outbreak data in the prospective surveillance setting, how will the Poisson and the negative binomial likelihood perform with specific spatiotemporal mean models and different amounts of previous data?
2. Comparison among the spatiotemporal mean models: Will adding spatial components regarding spatial random effects and neighborhood transmission terms be adequate for modeling epidemic outbreaks?

The rest of the paper is organized as follows. In the Methods section, we will introduce our Bayesian spatiotemporal models of interest. Mean models with various spatial characteristics and different epidemic transmission terms will be introduced. The negative binomial likelihood dispersion parameter is modeled using spatial and spatiotemporal effects. In the Results section, we will evaluate these models with the simulation data and the COVID-19 data of New Jersey (NJ) and South Carolina (SC). We analyzed the data with different mean models based on the Poisson and the negative binomial likelihoods with different window sizes. Discussion and conclusion of the results will follow in the last sections.

## Methods

### Spatiotemporal infectious disease models for the count data

In this paper, we are interested in the newly occurred infectious disease case count data collected from the specific spatial unit. These spatial units usually follow the government administrative boundaries such as states, counties, and census tracts. A Poisson likelihood and a negative binomial likelihood are commonly used for these types of count data. For these likelihoods, a log link is used to connect the covariates of interest and the count data. Mean ${\mu }_{ij}$ is modeled with linear model components using the log link.

1 $$\mathrm{log}\left({\mu }_{ij}\right)= {\mathrm{log}\left({S}_{ij}\right)+\alpha }_{0}+{u}_{i}+{v}_{i}+\mathrm{Epidemic\, component}$$

We assume a Susceptible-Infected-Removed (SIR) model [19] for the infectious disease mechanism. The particular version of the SIR model evaluated here was proposed by Morton and Finkenstädt [20] which was originally applied to the time series of the measles outbreak and extended in the spatiotemporal domain by Lawson and Song [21] for an influenza outbreak analysis. The offset term $\mathrm{log}\left({S}_{ij}\right)$ of the Eq. (1) was calculated using the SIR accounting equation. Define ${S}_{ij}$ as the size of the susceptible population at-risk at time i and area j. The accounting equation for the SIR model is

$${{S}_{ij }= S}_{i,j-1}-{y}_{i,j-1}-{R}_{i,j-1}.$$

The susceptible population for the current period j is derived as a subtraction of the previous disease counts $\left({y}_{i,j-1}\right)$ and removable population (${R}_{i,j-1}$) from the previous susceptible population. The equation considers the removed population ${R}_{i,j-1}$ caused by death, relocation, and other reasons. In this paper, we exclude the reinfected cases of COVID-19 in our SIR model. For COVID-19, although it is now commonly accepted that it is possible to be re-infected, we still do not fully understand the complex reinfection mechanisms, although loss of immunity and the emergence of new variants are important factors. Since our focus is the evaluation of the different spatiotemporal models rather than the precise mathematical formulation of infection mechanisms, such as estimating R-naught, our assumption does not prevent achieving the purpose of this paper. For our simulation, we assumed a rough 1% as the death rate based on the current total deceased population divided by the infected population from COVID-19 data (As of June 14^th^, 2022, US total death/US total cases = 1,008,116/85,570,063 = 0.012 from [22]).

When we recognize that the area-specific effects exist in our data, spatially correlated and uncorrelated random effect ${u}_{i}$ and ${v}_{i}$ for the area i can be used to specify the spatial characteristics. Besag et al. [23] first used both correlated and uncorrelated random-effects models with the normal prior distributions in the Bayesian hierarchical model as $\mathrm{log}\left({\theta }_{i}\right)= {\alpha }_{0}+{u}_{i}+{v}_{i}$, which is called Besag-York-Mollié (BYM) model. ${\theta }_{i}$ is the relative risk of area i, and disease cases for this model are assumed to follow the Poisson distribution. Spatial correlated random coefficient ${u}_{i}$ have the intrinsic conditional autoregressive (ICAR) model [23], which has the form

$${\mathrm{u}}_{\mathrm{i}}|{u}_{\left(i\right)}\sim N({\overline{u} }_{i},\frac{{\sigma }_{u}^{2}}{{n}_{{\delta }_{i}}}),$$

where ${\overline{u} }_{i}= \sum\nolimits_{j\in {\delta }_{i}}\frac{{u}_{j}}{{n}_{{\delta }_{i}}}, {\updelta }_{\mathrm{i}}$ = neighborhood of area i, ${u}_{\left(i\right)}$ = all regions except region i, and ${n}_{{\delta }_{i}}$ is the number of the neighborhood regions of area i. The spatial uncorrelated random component ${v}_{i}$ follows the zero-mean Gaussian prior distribution with the variance ${\sigma }_{v}^{2}$. We acknowledge that the variance term of spatial random components can capture the over-dispersion due to spatial characteristics. However, we also assume that over-dispersion of the infectious disease wave is affected by both space and time bounded characteristics. Therefore, comparing the negative binomial and Poisson likelihoods in Bayesian spatiotemporal infectious surveillance analysis can show i) whether a spatial random component is an effective way to capture the over-dispersion property of the data and ii) which characteristics of overdispersion are more important to model the infectious disease outbreaks.

In order to directly model the epidemic outbreak effect, we adopted the two-component endemic-epidemic model from [24] for our Bayesian spatiotemporal models. The authors divided the log-linear mean model into endemic and epidemic components. In our model, we capture two infectious diseases spread patterns: the lagged epidemic effect and the neighborhood epidemic effect. The lagged epidemic effect assumes that the current disease count is influenced by the previous time’s disease count pattern. Therefore, disease spread can be intensified in the area where we had more disease counts in the previous period. We model this pattern with the term ${\beta }_{ep1}\cdot \mathrm{log}({y}_{i,j-1})$. The neighborhood epidemic effect assumes that the outbreak starting in one area will spread first into its neighboring areas. We model this pattern with the summation term of neighboring areas’ disease counts ${\beta }_{ep2}\cdot \mathrm{log}(\sum\nolimits_{{\delta }_{i}}{y}_{{\delta }_{i},j-1})$. This effect was used in [21, 25], effectively incorporating the neighborhood transmission of infectious diseases.

The endemic component incorporates the infectious disease’s baseline transmission rate and the area’s characteristics. In the case of COVID-19, we do not know its baseline or future endemic characteristics yet. In this case, spatial random effects, as suggested by Besag-York-Mollié [23] can be used to accommodate the factors related to the endemic nature of the infectious disease.

In summary, the full model of our spatiotemporal mean model has the following form:

$$\mathrm{log}\left({\mu }_{ij}\right)=\mathrm{log}\left({S}_{ij}\right)+{\alpha }_{0}+{\beta }_{ep1}\cdot \mathrm{log}\left({y}_{i,j-1}\right)+{u}_{i}+{v}_{i}+{\beta }_{ep2}\cdot \mathrm{log}(\sum\limits_{{\delta }_{i}}{y}_{{\delta }_{i},j-1})$$

with the Poisson and the negative binomial likelihoods for the disease count.

The prior distributions for the intercept ${\alpha }_{0}$, regression parameters ${\beta }_{ep1}$ and ${\upbeta }_{\mathrm{ep}2}$ follow the conventional zero-mean normal distribution with variance ${\upsigma }_{0}^{2}$ for the intercept term, and shared ${\mathrm{\sigma }}_{\upbeta }^{2}$ for regression terms.

For variance terms, we further assume weakly-informative prior distributions, and standard deviations follow uniform prior distributions recommended by Gelman [26]. We also considered a different specification of the uniform priors, especially uniform shrinkage prior for the Poisson spatiotemporal model [27, 28] but selected the more general form of the uniform prior. Because our purpose is the comparison of different mean models and likelihoods while the length of previous data is fixed, we would like to use the prior for the standard deviation of each parameter to be used for both the Poisson and the negative binomial likelihoods and for other parameters as well as spatiotemporal components. Specifically we used the uniform prior [0,10] for the standard deviation of spatially correlated and uncorrelated random components, parameters for epidemic components ${(\beta }_{ep1},{\beta }_{ep2})$, and the parameter for the negative binomial dispersion component ${(\beta }_{r})$. For the prior of standard deviation of ${\alpha }_{0}$, we used Uniform [0,15]. We wanted to assume weakly informative prior for these standard deviation components for each parameter and the maximum variance of 100 and 225 satisfies our purpose compared to the average sampled values for each parameter during the simulation study and the COVID-19 analysis. Also, it provided the reasonable fit and convergence for these parameters.

For the negative binomial likelihood, there is a dispersion parameter for the over-dispersed data, and many previous analyses used the pre-specified gamma prior [8, 16]. However, we observe from the COVID-19 data that the degree of over-dispersion is different depending on specific time and areas. Modeling this varied dispersion parameter to specify the characteristics of the site was well established in the traffic accident literature [29, 30] and also recently used for the COVID-19 data analysis [15]. We assumed that dispersion of the infectious disease outbreak can be influenced by the area-specific characteristics and used the spatial correlated and uncorrelated random components from the mean model to specify the dispersion parameter. We evaluated two forms of dispersion parameter modeling: 1) dispersion parameter considers spatial characteristics $({r}_{i })$ and is modeled with spatial components ${u}_{i} \,and\, {v}_{i}$:

$$\mathrm{log}\left({\mathrm{r}}_{\mathrm{i}}\right)={r}_{0}+{u}_{i}+{v}_{i}$$

2) dispersion parameter considers the variation of spatial effect from its neighborhood and the difference of each time period ($${r}_{ij })$$:

$$\mathrm{log}\left({\mathrm{r}}_{\mathrm{ij}}\right)={r}_{0}+{u}_{i}+{v}_{i}+{\beta }_{r}\cdot \mathrm{log}(\sum\limits_{{\delta }_{i}}{y}_{{\delta }_{i},j-1})$$

Prior distributions for the intercept and regression parameter terms of the dispersion parameter are a zero-mean Gaussian distribution with the variance ${\upsigma }_{{\mathrm{r}}_{0}}^{2} \,and\, {\upsigma }_{{\upbeta }_{\mathrm{r}}}^{2}$ respectively.

### Model implementation

In this paper, we evaluated the performance of Bayesian spatiotemporal models for prospective surveillance by the sliding window approach. The models of interest are presented in Table 1. For each of the Poisson and the negative binomial likelihoods, we considered three different mean model forms. In the case of the negative binomial likelihood, for each mean model form, we further considered two different dispersion parameter forms. Each model in Table 1 was analyzed with 3 different window sizes.

**Table 1:** Bayesian spatiotemporal models considered for the evaluations

	Poisson	Negative binomial	
		$\mathrm{log}\left({r}_{i}\right)= {r}_{0}+{u}_{i}+{v}_{i}$	$\mathrm{log}\left({r}_{ij}\right)= {r}_{0}+{u}_{i}+{v}_{i}+{\beta }_{r}\bullet \mathrm{log}(\sum_{{\delta }_{i}}{y}_{{\delta }_{i},j-1})$
M1 $\mathrm{log}\left({\mu }_{ij}\right)=\mathrm{log}\left({S}_{ij}\right)+{\alpha }_{0}+{\beta }_{ep1}\cdot \mathrm{log}\left({y}_{i,j-1}\right)$	PM1 (Poisson likelihood with the mean model M1)	NBM1RS (Negative binomial likelihood with the mean model 1 and dispersion varied by area)	NBM1RST (Negative binomial likelihood with the mean model 1 and dispersion varied by area and time)
M2 $\mathrm{log}\left({\mu }_{ij}\right)=\mathrm{log}\left({S}_{ij}\right)+{\alpha }_{0}+{\beta }_{ep1}\cdot \mathrm{log}\left({y}_{i,j-1}\right)+{u}_{i}+{v}_{i}$	PM2	NBM2RS	NBM2RST
M3 $\mathrm{log}\left({\mu }_{ij}\right)=\mathrm{log}\left({S}_{ij}\right)+{\alpha }_{0}+{\beta }_{ep1}\cdot \mathrm{log}\left({y}_{i,j-1}\right)+{u}_{i}+{v}_{i}+{\beta }_{ep2}\cdot \mathrm{log}(\sum\limits_{{\delta }_{i}}{y}_{{\delta }_{i},j-1})$	PM3	NBM3RS	NBM3RST

The M1 model is the basic epidemic model without spatial components. It only depends on the lagged time series effect of previous disease counts. M2 is the infectious disease model with spatial correlated and uncorrelated random effects (BYM model) with the lagged epidemic term. In model M3, we added the neighborhood epidemic effect terms to the model M2. For the negative binomial likelihood, we considered both ${\mathrm{r}}_{\mathrm{i}}$ (dispersion due to a spatial effect) and ${\mathrm{r}}_{\mathrm{ij}}$ (dispersion due to a spatiotemporal effect) as dispersion parameter specifications.

We first evaluated these models with simulated data and then applied them to the real COVID-19 data to assess their model performances. We conducted the posterior sampling with the mixed Gibbs-Metropolis algorithms using the ‘nimble’ package (version 0.11.1) [31] in R. The details of the full Bayesian implementation are in Additional file 1.

### Model performance evaluation criteria

Model performance is evaluated by the deviance information criterion (DIC), the mean squared error (MSE), and the mean absolute one-step-ahead prediction error (MAOSPE). DIC is the widely used goodness of fit measure in Bayesian analysis. It is defined as

$$\mathrm{DIC}=2{\mathrm{E}}_{\uptheta |\mathrm{y}}\left(D\right)-D[{\mathrm{E}}_{\uptheta |\mathrm{y}}\left(\theta \right)]$$

where D[.] is the deviance of the model and $\uptheta$ is the parameter set of the model. DIC is based on the comparison of the average deviance and the deviance of the posterior parameter estimates [32]. For model comparison, smaller DIC shows better goodness of fit.

MSE is the comparison of the data point and posterior mean estimate.

$$\mathrm{MSE}= {\sum }_{i=1}^{M}{\sum }_{j=1}^{t}{\sum }_{s=1}^{S}{\left({y}_{ij}-{\widehat{\mu }}_{ijs }\right)}^{2}/(M\cdot t\cdot S),$$

where S is the number of MCMC samples, M is the total number of the area, and t is the total number of time periods. It was calculated as the sum of the squared differences between actual data ${y}_{ij}$ and posterior estimates at each sampling period $({\widehat{\mu }}_{ijs})$ and subsequently took the average of the summed value over the whole time, space and sampling periods.

For evaluating short-term prediction, we generated the one-step-ahead prediction from the posterior estimates of the last time period and compared the value with real data.

$$\mathrm{MAOSPE }= {\sum }_{i=1}^{M}{\sum }_{j=1}^{t}{\sum }_{s=1}^{S}|{y}_{i,j+1}-{\widehat{y}}_{i,j+1,s}|/(M\cdot t\cdot S)$$

For each sampling period s, ${\widehat{y}}_{i,j+1,s}$ was sampled from the estimated, ${\widehat{\mu }}_{i,j+1,s}$ which was calculated using the values from the previous time period j.

### Simulation method

We conducted simulation studies to evaluate how each model in Table 1 performs for different prospective surveillance settings. Specifically, we would like to know how each spatiotemporal surveillance model performs depending on different window sizes.

**Fig. 1:**
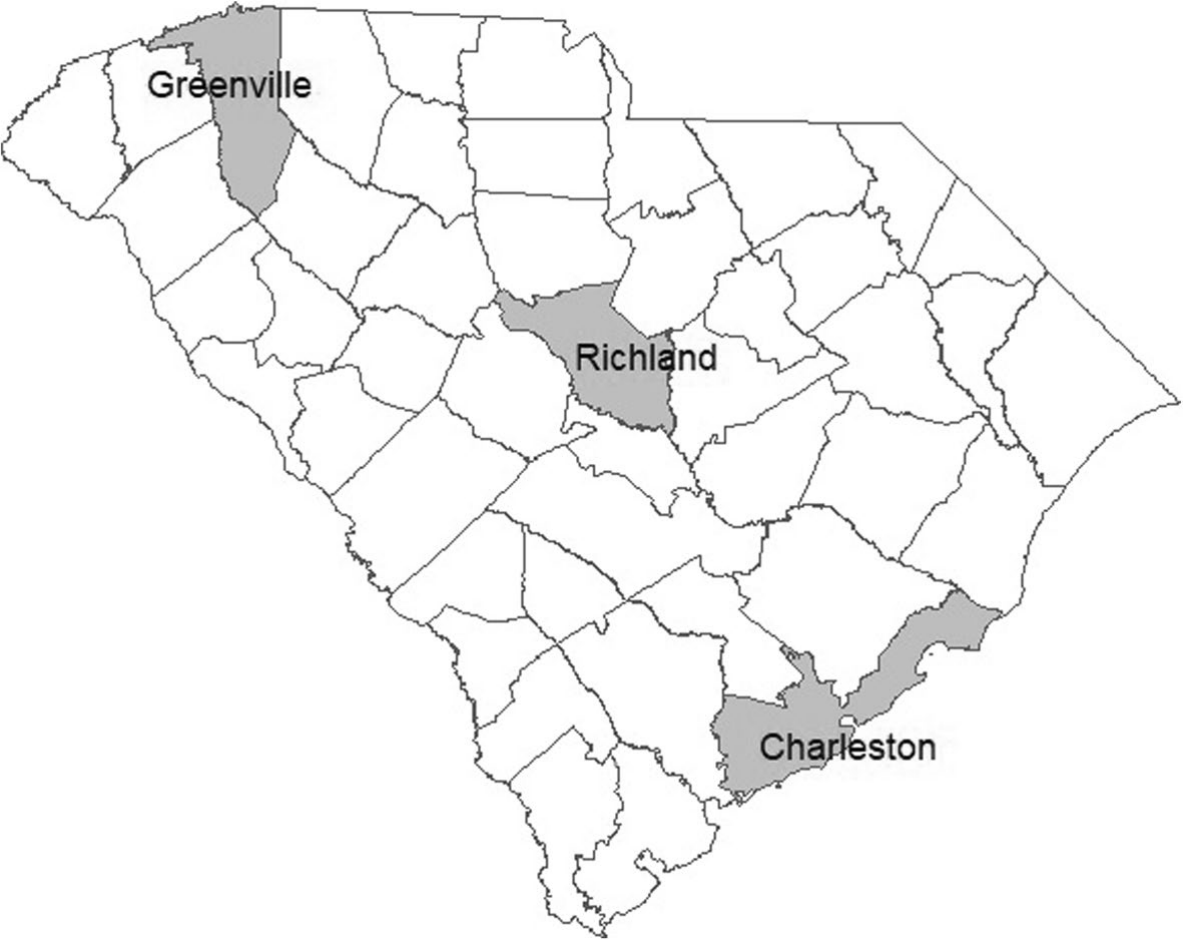
The map of South Carolina with 3 highlighted metropolitan counties, which was used for simulation data generation

**Fig. 2:**
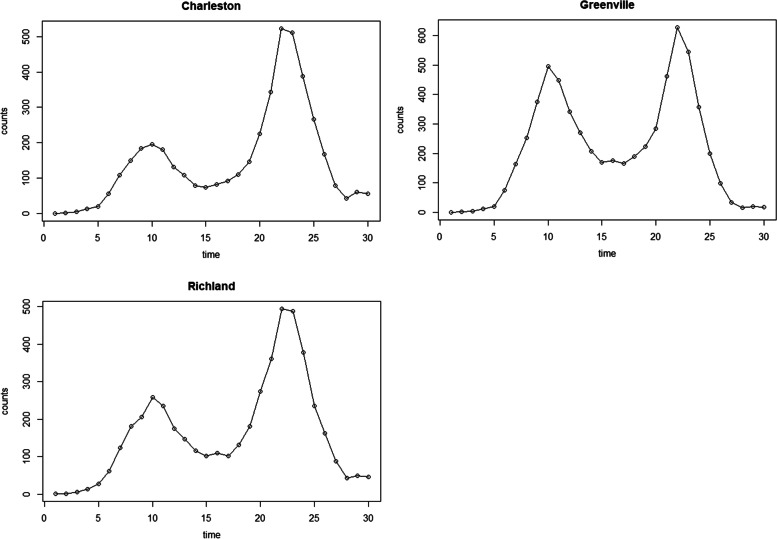
Example of the simulation data for 3 metropolitan areas of the South Carolina

To mimic the real surveillance data, the South Carolina (SC) map was used for our simulation analysis. As we see from Fig. 1, it has 3 distinct metropolitan areas (Greenville, Richland, and Charleston) from northwest to southeast. We considered 30 time periods and assigned two disease outbreak waves, one from 6 to 15 and another from 18 to 26. We assumed the outbreak started in 3 metropolitan areas and spread through neighboring counties. The disease count during the epidemic was simulated following CDC guidelines [33]. Before the epidemic, disease counts were kept to less than 10 cases per 100,000 populations (low-risk level). Upon the start of the epidemic, it is assumed to increase to more than 50 cases per 100,000 populations (above the ‘high’ risk level). Figure 2 shows the example of the simulated data for 3 metropolitan counties, and the details of the data generation process are provided in Additional file 2. For the window length, we considered 30, 7, and 3 time periods. The window size of 30 uses the entire data, which is the same as conducting the retrospective analysis. Window size 7 was chosen because it is long enough to cover the simulated outbreak wave’s entire increasing or decreasing trend. A window size of 3 represents the situation when a very current trend is only included for our surveillance. We calculated each window segment’s DIC, MSE, and short-term prediction performance. For MAOSPE, we sampled the one-step-ahead prediction for each window analysis result and compared them to the data in the following time period. Each model in Table 1 was fitted for the different window lengths of 30, 7, and 3 for 100 simulated data sets. For each analysis, 500 samples were produced after 50,000 iterations with 45,000 burn-in periods and thinning by 10.

## Results

### Simulation study

We first reported the analysis results with the simulated data as explained in Simulation method section. The analysis results for each model with the different window sizes are presented in Tables 2, 3 and 4 and Additional file 3 Tables A1-A4. First, we compared how each model performs in the descriptive analysis setting (retrospective analysis) using a whole 30-time period analysis.

**Table 2:** DIC and MSE values from the analysis of the window size = 30

	DIC	MSE
PM1	14,542	727
PM2	10,892	640
PM3	10,882	653
NBM1RS	8051	1650
NBM1RST	7967	1501
NBM2RS	7532	540
NBM2RST	7554	535
NBM3RS	7297	606
NBM3RST	7302	595

**Table 3:** DIC, MSE, and MAOSPE for the simulation analysis for the window size 7 when time 19 to 25 (fluctuation trend)

	**DIC**	**MSE**	**MAOSPE**
PM1	3786	1200	27.02
PM2	3097	977	25.76
PM3	3109	1001	27.09
NBM1RS	2283	2011	36.32
NBM1RST	2279	2071	36.67
NBM2RS	2254	1140	42.76
NBM2RST	2264	1151	42.78
NBM3RS	2188	1408	38.58
NBM3RST	2189	1429	38.90

**Table 4:** DIC, MSE, and MAOSPE for the simulation analysis for the window size 3 when time 21 to 23 (fluctuation trend)

	**DIC**	**MSE**	**MAOSPE**
PM1	1256	625	25.18
PM2	1024	358	26.14
PM3	1020	430	27.70
NBM1RS	982	1302	37.67
NBM1RST	944	852	38.74
NBM2RS	978	532	35.51
NBM2RST	990	516	35.69
NBM3RS	946	859	37.24
NBM3RST	951	1008	37.63

Table 2 shows the DIC and MSE performance of each of the Poisson and the negative binomial models. The data of the window length 30 are over-dispersed, including two high-risk outbreak waves (more than 50 per 100,000 cases). Negative binomial likelihoods show better model fits than Poisson models, as shown by comparing DIC with 10882 (PM3) and 7302 (NBM3RST). As we expected from the role of spatial random coefficients, the models with spatial correlated and uncorrelated components (M2 and M3) show better performances than the models only with a lagged time effect (M1). Models M2 and M3 can follow epidemic transmissions due to both the neighborhood effect and the disease transmission of the previous time, so they showed better goodness of fit results and smaller MSE than model M1. Between the mean model M2 and M3, the mean model with neighborhood epidemic effects (M3) shows better goodness of fit compared to the model without it (M2).

Table 3 and Additional file 3 Tables A1–A2 present DIC, MSE, and MAOSPE results for window size 7. For each time period, the sliding window size of 7 includes 6 previous time periods and current disease cases. One benefit of this setting is that we can investigate the model performance of the different disease trends. Our simulation data was designed to have 2 infectious disease outbreak waves, and each wave was set for 15 time periods. A window size of 7 can capture increasing or decreasing trends or include fluctuation between trends. In order to present each model systematically, we selected the time segment from the different locations: 1) increasing, 2) decreasing, and 3) fluctuating (changing from increasing to decreasing trend) trends.

Table 3 presents the goodness of fit result for the window size of 7 when the disease trend fluctuates. It shows that the negative binomial models show smaller DIC values than the Poisson models. The mean model M3 shows the smallest DIC values for both Poisson and negative binomial likelihoods. However, each model’s MSE result differs depending on the trend. For the increasing and decreasing trends, the NBM3RST model has the smallest MSE, but for the fluctuation trend, the mean model PM2 has the smallest MSE value. For window sizes 7 and 3, we can evaluate the short-term prediction result using the data from the next time period. The MAOSPE column shows that PM2 and PM3 models show smaller MAOSPE values than NB models.

Lastly, we evaluated the models with a window size of 3. When the transmission rate is very high, a window size of 3 can also have a large variability, but otherwise, it can be less variable compared to the window size of 7 and 30. Table 4 and Additional file 3 Tables A3-A4 show the results from the analysis of window size 3. Table 4 shows that negative binomial models provide a smaller DIC than Poisson models, but the difference is narrower between the negative binomial and the Poisson likelihood models compared to the longer window size results. MSE and MAOSPE results show that when infection trends are increasing, NB and Poisson models are comparable. However, Poisson models show smaller MSE and MAOSPE when infection trends are decreasing or fluctuating.

In summary, we examined each model with different simulation settings. We found that (i) the mean models with spatial random coefficients (M2 and M3) provide a smaller DIC value compared to the models with only previous epidemic components (M1); (ii) the negative binomial models provide a better model fit when we have a longer window size but their difference becomes smaller when we have a shorter window size; and iii) overall, the Poisson models provide smaller MSE and MAOSPE values when we use the window size of 7 and 3.

### Real data analysis of COVID-19

Next, we shifted our focus to real outbreak data analysis of the COVID-19 pandemic.

**Fig. 3:**
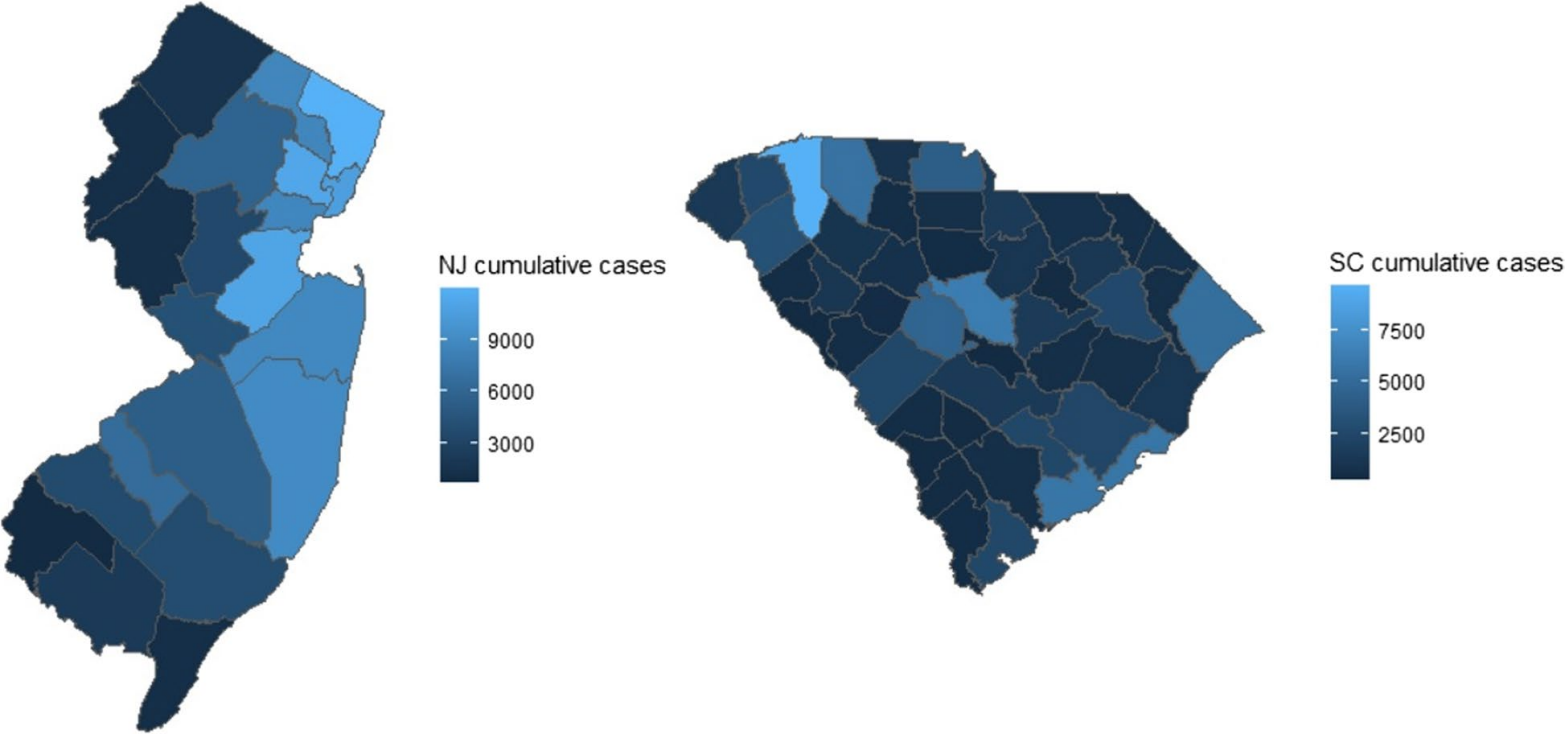
Maps of NJ and SC with cumulative COVID-19 cases from March 2020 to Feb 2021

We used the data from the state health departments collected in the New York Times GitHub [34] from March 15^th^, 2020 up to 52 weeks and investigated the data of COVID-19 for the states of New Jersey and South Carolina. Figure 3 shows each county’s cumulative COVID-19 cases of the study period. The state of New Jersey was selected for the comparison to South Carolina since it is the state with the highest population density (NJ: 1263.2/sq.mile and SC: 170.2/sq.mile in census 2020 [35]) with a quite homogeneous population number in each county. New York Times GitHub provides the daily updated data for each county based on the reporting of each state health authority. The daily numbers from the national database allow us to track the spread of COVID-19 at the finest level. However, it suffers from large fluctuations due to random noise and systematic problems such as delayed reporting and a weekly pattern due to artifacts of the reporting schedule. Since our purpose is the evaluation of different models in the real data, we used the weekly average of the daily data to prevent these systematic problems and allow us to track the pattern of disease spread more clearly and with less random noise. For the first year, most of the SC area showed average case counts of less than 50 cases per 100,000 residents for 28 days. On the contrary, the first wave of NJ showed a very high transmission rate. Therefore we could compare the models with different transmission levels by comparing NJ and SC.

In the simulation analysis, we evaluated the various mean models for the Poisson and the negative binomial likelihood. Based on the simulation results, we used the mean model M3 for the real data analysis since M3 showed the best goodness of fit performance in the simulation study. The models evaluated in this section are summarized in Table 5. We implemented MCMC analysis with the R package Nimble [31] and sampled from 100,000 iterations with 90,000 burn-in periods and thinned the sample by 10. We used the same prior specifications for real data analysis as the simulation analysis. We used ICAR distribution for ${u}_{\mathrm{i}}$, and the zero-mean normal distribution was used for ${v}_{\mathrm{i}},{ \alpha }_{0},{ \beta }_{r}{, \beta }_{ep1},{ \beta }_{ep2},$ and ${r}_{0}$. For variance terms, uniform (0,10) priors were used for the standard deviation. We used the window size of 52 (whole year data), 7, and 3 weeks to compare the effect of window size on the goodness of fit and prediction performance.

**Table 5:** Summary of the models to be evaluated

PM3 $$\mathrm{log}\left({\mu }_{ij}\right)=\mathrm{log}\left({S}_{ij}\right)+{\alpha }_{0}+{\beta }_{ep1}\cdot \mathrm{log}\left({y}_{i,j-1}\right)+{u}_{i}+{v}_{i}+{\beta }_{ep2}\cdot \mathrm{log}(\sum\limits_{{\delta }_{i}}{y}_{{\delta }_{i},j-1})$$
NBM3RS $$\mathrm{log}\left({\mu }_{ij}\right)=\mathrm{log}\left({S}_{ij}\right)+{\alpha }_{0}+{\beta }_{ep1}\cdot \mathrm{log}\left({y}_{i,j-1}\right)+{u}_{i}+{v}_{i}+{\beta }_{ep2}\cdot \mathrm{log}(\sum\limits_{{\delta }_{i}}{y}_{{\delta }_{i},j-1})$$ $$\mathrm{log}\left({r}_{i}\right)= {r}_{0}+{u}_{i}+{v}_{i}$$
NBM3RST $$\mathrm{log}\left({\mu }_{ij}\right)=\mathrm{log}\left({S}_{ij}\right)+{\alpha }_{0}+{\beta }_{ep1}\mathrm{log}\left({y}_{i,j-1}\right)+{u}_{i}+{v}_{i}+{\beta }_{ep2}\cdot \mathrm{log}(\sum\limits_{{\delta }_{i}}{y}_{{\delta }_{i},j-1})$$ $$\mathrm{log}\left({r}_{ij}\right)= {r}_{0}+{u}_{i}+{v}_{i}+{\beta }_{r}\cdot \mathrm{log}(\sum\limits_{{\delta }_{i}}{y}_{{\delta }_{i},j-1})$$

As in the case of the simulation section, we compared the goodness of fit by DIC, posterior mean estimation by MSE, and short-term prediction performance evaluated by the MAOSPE measure. Table 6 presents the analysis results of the 52 time periods.

**Table 6:** DIC, MSE and MAOSPE table for the analysis of NJ and SC with the window size of 52 weeks

	NJ			SC		
	DIC	MSE	MAOSPE	DIC	MSE	MAOSPE
PM3	25,527.52	4235.22	18.96	16,340.29	196.37	4.71
NBM3RS	12,033.40	4676.00	124.49	14,453.65	207.48	34.60
NBM3RST	10,107.35	4653.78	109.57	14,277.01	207.78	36.44

First, we compare the goodness of fit measures. From the comparison of the negative binomial and the Poisson likelihoods, the negative binomial likelihood models show smaller DIC values than the PM3. With one whole year of data, the negative binomial likelihood better accommodates the variation of the data with its dispersion parameter. The difference between the negative binomial and Poisson likelihoods is significantly larger for the NJ result since NJ data showed very high variability compared to the moderate transmission rate of SC data. On the contrary, the short-term prediction and MSE for the window length of 52 show a different pattern with the goodness of fit measure. MSE and MAOSPE columns of Table 6 show that the PM3 has smaller MSE and MAOSPE values than the negative binomial models.

Next, we investigated the window length of 7 during the 52-week periods. Identical to the simulation result, we presented the window analysis results categorized by the outbreak trend in three parts: increasing, decreasing, and changing trends (fluctuation). For this purpose, we selected the time segment 33–39 weeks (NJ) and 11–17 weeks (SC) for increasing trend, 9–15 (NJ) and 20–26 (SC) for decreasing trend, and 4–10 (NJ) and 16–22 (SC) for the fluctuating trend. Table 7 and Additional file 3 Tables A5-A6 show the results of the window size 7. The results are similar pattern compared to the one-year analysis. The negative binomial shows better goodness of fit, but the PM3 model shows the more stable estimation of posterior mean and predictive values shown by smaller MSE and MAOSPE values.

**Table 7:** DIC, MSE and MAOSPE table for the analysis of NJ and SC for the window size of 7 (t = 4–10 weeks (NJ) and t = 16–22 weeks (SC) with fluctuation trend)

	NJ			SC		
	DIC	MSE	MAOSPE	DIC	MSE	MAOSPE
PM3	2512.20	870.42	13.36	2259.62	101.67	6.76
NBM3RS	1355.98	1423.24	46.94	2099.09	163.03	18.63
NBM3RST	1364.13	1300.83	43.82	2073.57	149.33	18.69

Window size of 3 shows a different pattern from the result of the long window size of 7 and 30. From Table 8, Additional file 3 Tables A7 and A8, we can observe that DIC values of PM3 are smaller than NBM3RS and NBM3RST when NJ has increasing and fluctuating trends and SC has a decreasing trend. MSE and MAOSPE tables show that PM3 has smaller MSE and MAOSPE values than its NB counterparts. The window size of 3 has less variability in the data, therefore, the difference among the 3 models is smaller than the results of longer window sizes, and PM3 shows a better model fit in some cases. Overall, the PM3 shows smaller or comparable DIC values and stable MSE and short-term prediction performance than NBM3RS and NBM3RST.

**Table 8:** DIC, MSE and MAOSPE table for the analysis of NJ and SC for the window size of 3 (t = 7–9 weeks (NJ) and t = 18–20 weeks (SC) with fluctuation trend)

	NJ			SC		
	DIC	MSE	MAOSPE	DIC	MSE	MAOSPE
PM3	512.29	89.46	12.27	910.43	50.09	10.94
NBM3RS	572.12	356.31	37.59	897.27	61.05	13.68
NBM3RST	527.08	106.95	48.72	900.27	61.92	13.65

## Discussion

In this paper, we evaluated Bayesian spatiotemporal infectious disease models in the prospective surveillance setting with simulation data and real COVID-19 data analysis. Prospective surveillance demands good performance in short-term prediction and the adjustment to the different past data lengths. We investigated how spatiotemporal mean models performed with two count data likelihoods of the Poisson and the negative binomial likelihoods and with different sliding window lengths. Our results show that the choice of different window sizes affects the surveillance model performance. Adjusting window size for the prospective surveillance can be the balancing problem to achieve both stable prediction and removal of out-of-date information. Using a too short window size can make our statistical estimates unstable. However, using a too long window can make our current parameter estimates over-smoothed and unable to change quickly to reflect the current trend. Especially, COVID-19 data shows the pattern of waves, and each wave has a distinct shape and intensity as we go through it. For example, there exists a difference among the first wave, the wave caused by the Delta variant and the wave caused by Omicron variant. Hence, we needed to use our past wave data, but quickly update the information at hand to adapt to the current wave. Our simulation and COVID-19 data analysis showed that a window size of at least 7-time points is preferred for our negative binomial models, including dispersion parameter modeling and spatial random components. With a longer data size, the negative binomial likelihood-based model provides stable estimation and better goodness of fit than Poisson likelihoods. Also, our data analysis results show that the negative binomial spatiotemporal models work well with data with large variabilities, such as the data with more than 50 cases per 100,000 infection rate. With small variabilities such as the window size 3, negative binomial and Poisson based models are comparable, and Poisson likelihood-based models provide stable prediction and sometimes better goodness of fit. Therefore, our preference for a specific model in the prospective surveillance can be decided according to the research goal of the model. Better goodness of fit performance for the large wave, such as NJ data for the window size 52 and 7, clearly shows where the strength of the negative binomial likelihood-based models exists. When we have over-dispersed data and long periods of past data are available and necessary for our purpose, the negative binomial likelihood provides the appropriate model fit. However, the difference among our likelihood-based models was reduced when the window size decreased. Even though we have the over-dispersed data, if a short window length is appropriate for our surveillance, then Poisson likelihood-based models provide comparable goodness of fit, posterior mean estimates, and prediction results with smaller error metrics. As we show in the window size of 3, if we use the short length of past data and our data has less than moderate risk level, then Poisson likelihood is the parsimonious data model with stable prediction and the model fit.

We also evaluated different Bayesian spatiotemporal mean models and dispersion models for the prospective surveillance setting. Clearly, the models with spatial components (Models M2 and M3) perform better compared to the model with only lagged time effects from the previous time period of disease cases. The difference between the models M2 and M3 is not prominent in our analysis. The effect of the neighborhood epidemic effect is not apparent in our simulation, and it depends on trend, window size and statistical models. Since spatial random components also contain some of the neighborhood effects, the part of the neighborhood epidemic effect term of M3 is included in the model M2, and it can reduce the difference between M2 and M3 in our study. We also explore the effect of spatially or spatio-temporally modeled dispersion parameters in the spatiotemporal surveillance models. Similar to the neighborhood epidemic effect, the difference between the two dispersion models depends on the size of the disease outbreak and the disease trend.

Even though we conducted a thorough investigation of the performance of spatiotemporal infectious disease outbreak models, some limitations still exist for our study and future work might be needed. First, our simulation setting and COVID-19 data analysis were based on the knowledge available from 2020 to 2022. Our results can be changed depending on the characteristics of different infectious diseases such as transmission method, asymptomatic transmission, and vaccination rate. Second, future work can be done to investigate the effect of the different window sizes thoroughly. In this paper, we focused on comparing the different models, depending on the different window lengths. Another interesting study will evaluate the different window sizes for the same Bayesian spatiotemporal model. It can show the effect of long or short past data on parameter estimation stability and prediction accuracy for the infectious disease wave data.

Third, future work can be done to refine our Bayesian spatiotemporal infectious disease models. This paper modeled the dispersion parameter of the negative binomial models with spatial random components and neighborhood terms to accommodate spatiotemporal characteristics of the infectious disease data. Not many studies have been done about Bayesian spatiotemporal modeling with a negative binomial likelihood, so more interesting ways to model the dispersion parameter can be developed in the future. For the infectious disease modeling components, we can extend our epidemic terms to include more ‘trend’ parameters to accommodate the trend change and the volatility of the data. There is a growing body of infectious disease modeling forecasting analysis based on volatility and fluctuation [36]. Future work can be performed to quantify and consider the volatility within our Bayesian spatiotemporal infectious disease models. Also we can extend our SIR and endemic components to include the characteristics of infectious disease transmission. Many experts predict that COVID-19 will possibly stay with us as an endemic disease [37, 38]. As our knowledge grows, we can refine our “endemic” component of our model to accommodate endemic COVID-19. Currently, we modeled it with random effect components only following the area’s characteristics.

Finally, in this paper, we evaluated our models by the traditional summary metrics of forecasting such as MAOSPE and MSE. Future work could extend the model evaluation metrics to measure ‘probabilistic’ forecasting performance. There is a significant body of literature about evaluating the ‘probabilistic’ nature of forecasting activity. Gneiting [39] stated that all forecasting activities need to reflect the innated uncertainty and this uncertainty needs to be evaluated through the forecasting metric. These measures aim to evaluate the statistical consistency between probabilistic forecasts and real data. The logarithmic score [40] and the Dawid-Sebastiani score [41] are examples of these performance evaluation metrics (Gneiting et al. called them as ‘proper scoring rule’). Czado et al. [42] and Wei and Held [43] extended and presented these evaluation tools and statistical significance tests for the count data model. A comparison of Bayesian hierarchical spatial-temporal models regarding probabilistic predictive performance will be another exciting extension of this paper.

## Conclusion

We evaluated Bayesian spatiotemporal models of the infectious disease outbreak in the prospective surveillance. The spatiotemporal mean models based on the Poisson and the negative binomial likelihoods were evaluated. The negative binomial model showed better goodness of fit performance for the over-dispersed infectious disease outbreak data, especially for the longer window size. Poisson likelihood based models show more stable posterior estimates and short-term prediction performance, and their goodness of fit is as good as the negative binomial models when we analyze the short window length. The choice of the window size is important for the data model choice, and the choice of the window depends on how much variability our outbreak situation has. If we have highly volatile data in a short period of time, then our model selection will be different from the low volatility situation.

Through COVID-19 surveillance, public health entities highlighted the necessity and importance of prospective surveillance in the United States. The Center for Disease Control (CDC) announced the establishment of the new federal-level disease forecasting center [44] which will emphasize not only comprehensive data collection but also research on the inference and forecast of infectious disease outbreaks. We believe that this study will help infectious disease modelers, epidemiologists, and public health researchers improve infectious disease surveillance, including but not limited to the COVID-19 pandemic.

## Supplementary Information

**Additional file 1.** Details of the Bayesian implementation.

**Additional file 2.** Details of the simulation data generation.

**Additional file 3: Additional tables for the Results section. Table A1.** DIC, MSE and MAOSPE for the simulation analysis for the window size 7 when time 15 to 21 (Increasing trend). **Table A2.** DIC, MSE and MAOSPE for the simulation analysis for the window size 7 when time 22 to 28 (Decreasing trend). **Table A3.** DIC, MSE and MAOSPE for the simulation analysis for the window size 3 when time 17 to 19 (Increasing trend). **Table A4.** DIC, MSE and MAOSPE for the simulation analysis for the window size 3 when time 23 to 25 (Decreasing trend). **Table A5.** DIC, MSE and MAOSPE table for the analysis of NJ and SC for the window size of 7 (t = 33–39 weeks (NJ) and t = 11–17 weeks (SC) with increasing trend). **Table A6.** DIC, MSE and MAOSPE table for the analysis of NJ and SC for the window size of 7 (t = 9–15 weeks (NJ) and t = 20–26 weeks (SC) with decreasing trend). **Table A7.** DIC, MSE and MAOSPE table for the analysis of NJ and SC for the window size of 3 (t = 33–35 weeks (NJ) and t = 13–15 weeks (SC) with increasing trend). **Table A8.** DIC, MSE and MAOSPE table for the analysis of NJ and SC for the window size of 3 (t = 9–11 weeks (NJ) and t = 22–24 weeks (SC) with decreasing trend).

## Data Availability

The dataset used in the study is publicly available. COVID-19 Data are available from a GitHub repository: https://github.com/nytimes/covid-19-data.

## Abbreviations

MCMC: Markov Chain Monte Carlo
DIC: Deviance information criteria
MSE: Mean square error
MAOSPE: Mean absolute one-step ahead prediction error
M1: Mean model 1
M2: Mean model 2
M3: Mean model 3
PM1: Poisson likelihood with the mean model 1
PM2: Poisson likelihood with the mean model 2
PM3: Poisson likelihood with the mean model 3
NBM1RS: Negative binomial likelihood with the mean model 1 and dispersion varied by area
NBM1RST: Negative binomial likelihood with the mean model 1 and dispersion varied by area and time
NBM2RS: Negative binomial likelihood with the mean model 2 and dispersion varied by area
NBM2RST: Negative binomial likelihood with the mean model 2 and dispersion varied by area and time
NBM3RS: Negative binomial likelihood with the mean model 3 and dispersion varied by area
NBM3RST: Negative binomial likelihood with the mean model 3 and dispersion varied by area and time
