# Supplementary material for: Evaluation of Bayesian spatiotemporal infectious disease models for prospective surveillance analysis
Source: BMC Med Res Methodol. 2023 Jul 22;23:171. doi: 10.1186/s12874-023-01987-5 (PMC10363300; doi:10.1186/s12874-023-01987-5)
Supplement: Supplementary file 1 — Additional file 1. Details of the Bayesian implementation. [file 12874_2023_1987_MOESM1_ESM.pdf]

Additional File 1. Details of the Bayesian implementation

Full Bayesian analysis was conducted on our hierarchical Bayesian spatio-temporal models. Prior distributions were specified as explained in section 2.1. The joint posterior distribution in the case of the negative binomial disease count model is proportional to the product of the likelihood function and the prior distributions.

$$p\left(u_i, v_i, \beta_{ep1}, \beta_{ep2}, \beta_r, \alpha_0, r_0, \sigma_0^2, \sigma_{r0}^2, \sigma_u^2, \sigma_v^2, \sigma_\beta^2, \sigma_r^2 | \boldsymbol{y_{t-l+1}}, \boldsymbol{y_{t-l+2}}, \cdots \boldsymbol{y_t}\right)$$

$$\propto \prod_{i=1}^{M} \prod_{j=t-l+1}^{t} \binom{y_{ij} + r_{ij} - 1}{y_{ij}} \cdot \left(\frac{r_{ij}}{\mu_{ij} + r_{ij}}\right)^{r_{ij}} \cdot \left(\frac{\mu_{ij}}{\mu_{ij} + r_{ij}}\right)^{y_{ij}}$$

$$\times \sigma_u^{-M} \cdot \exp\left\{-\frac{1}{2\sigma_u^2} \sum_{i=1}^{M} \sum_{\delta_I} \left(u_i - u_j\right)^2\right\}$$

$$\times \sigma_v^{-M} \cdot \exp\left\{-\frac{1}{2\sigma_v^2} \sum_{i=1}^{M} v_i^2\right\}$$

$$\times \sigma_0 \cdot \exp\left\{-\frac{1}{2\sigma_0^2} \alpha_0^2\right\} \times \sigma_{r0} \cdot \exp\left\{-\frac{1}{2\sigma_{r0}^2} r_0^2\right\}$$

$$\times \sigma_\beta \cdot \exp\left\{-\frac{1}{2\sigma_\beta^2} \beta_{ep1}^2\right\} \times \sigma_\beta \cdot \exp\left\{-\frac{1}{2\sigma_\beta^2} \beta_{ep2}^2\right\} \times \sigma_r \cdot \exp\left\{-\frac{1}{2\sigma_r^2} \beta_r^2\right\}$$

$$\times f(\sigma_0^2) \times f(\sigma_{r0}^2) \times f(\sigma_u^2) \times f(\sigma_v^2) \times f(\sigma_\beta^2) \times f(\sigma_r^2) \qquad (2)$$

Equation (2) shows the joint posterior specification in the case of the negative binomial distribution. We adopted the sliding window approach to implementing prospective surveillance analysis. In our joint probability distribution (2), l is the pre-specified window length, so we will use the data from the time $\mathrm{t} - l + 1$ to t. This enables us to discard out-of-date information, and also it maintains the number of parameters fixed as the time period increases. The joint posterior distribution cannot be

calculated analytically, so we sampled the posterior distribution using Markov chain Monte Carlo (MCMC) simulation methods.
