# Supplementary material for: Evaluation of Bayesian spatiotemporal infectious disease models for prospective surveillance analysis
Source: BMC Med Res Methodol. 2023 Jul 22;23:171. doi: 10.1186/s12874-023-01987-5 (PMC10363300; doi:10.1186/s12874-023-01987-5)
Supplement: Supplementary file 2 — Additional file 2. Details of the simulation data generation. [file 12874_2023_1987_MOESM2_ESM.pdf]

Additional File 2. Details of the simulation data generation

For the disease count data generation, prior for each parameter was set based on the posterior estimation of the COVID-19 data analysis from New Jersey and South Carolina. During the epidemic period, the transmission rate of three populous counties increased. We implemented this increase in transmission by multiplying 1.5 for calculated μ_{ij} when the disease outbreak starts. So these areas have a higher risk than other counties in SC for the epidemic periods. The data was generated from the mean model 3 from Table 1.

$$\log(\mu_{ij}) = \alpha_0 + u_i + v_i + \beta_{ep1} \cdot y_{i,j-1} + \beta_{ep2} \cdot \sum_{\delta_i} y_{\delta_i,j-1}$$

We need to generate the over-dispersed count data with varied dispersion as the time and the area change. In order to generate controlled over-dispersed data, we set the total disease counts for each time period to accommodate highly infectious disease waves and sampled through a Poisson-multinomial relationship. In each time period, we sampled the disease count through multinomial distribution conditioning on the total counts n_j for the time period j.

$$(y_{1j}, y_{2j} \dots y_{Mj)} = multinomial\ (p_{1j}, p_{2j} \dots, p_{ij}, n_j),$$

where $p_{ij} = \frac{\mu_{ij}}{\sum_i \mu_{ij}}$, n_j is the fixed disease count for the time j and varied for each time period.

Over-dispersed total disease counts for each time period and spatial correlated random components enable us to simulate the data with dispersion varied in each time and area. We generated 100 separate datasets for simulation to consider the random variability.
