# Supplementary material for: Evaluation of Bayesian spatiotemporal infectious disease models for prospective surveillance analysis
Source: BMC Med Res Methodol. 2023 Jul 22;23:171. doi: 10.1186/s12874-023-01987-5 (PMC10363300; doi:10.1186/s12874-023-01987-5)
Supplement: Supplementary file 3 — Additional file 3: Additional tables for the Results section. Table A1. DIC, MSE and MAOSPE for the simulation analysis for the window size 7 when time 15 to 21 (Increasing trend). Table A2. DIC, MSE and MAOSPE for the simulation analysis for the window size 7 when time 22 to 28 (Decreasing trend). Table A3. DIC, MSE and MAOSPE for the simulation analysis for the window size 3 when time 17 to 19 (Increasing trend). Table A4. DIC, MSE and MAOSPE for the simulation analysis for the window size 3 when time 23 to 25 (Decreasing trend). Table A5. DIC, MSE and MAOSPE table for the analysis of NJ and SC for the window size of 7 (t = 33–39 weeks (NJ) and t = 11–17 weeks (SC) with increasing trend). Table A6. DIC, MSE and MAOSPE table for the analysis of NJ and SC for the window size of 7 (t = 9–15 weeks (NJ) and t = 20–26 weeks (SC) with decreasing trend). Table A7. DIC, MSE and MAOSPE table for the analysis of NJ and SC for the window size of 3 (t = 33–35 weeks (NJ) and t = 13–15 weeks (SC) with increasing trend). Table A8. DIC, MSE and MAOSPE table for the analysis of NJ and SC for the window size of 3 (t = 9–11 weeks (NJ) and t = 22–24 weeks (SC) with decreasing trend). [file 12874_2023_1987_MOESM3_ESM.pdf]

Additional File 3. Additional tables for the Results section

Table A1. DIC, MSE and MAOSPE for the simulation analysis for the window size 7 when time 15 to 21 (Increasing trend)

	DIC	MSE	MAOSPE
PM1	2970	438	45.89
PM2	2008	96	13.34
PM3	1880	65	18.25
NBM1RS	1882	478	45.15
NBM1RST	2065	1226	43.38
NBM2RS	1949	398	43.06
NBM2RST	1948	404	44.24
NBM3RS	1751	98	57.94
NBM3RST	1744	90	61.23

Table A2. DIC, MSE and MAOSPE for the simulation analysis for the window size 7 when time 22 to 28 (Decreasing trend)

	DIC	MSE	MAOSPE
PM1	6406	1544	13.21
PM2	3892	338	6.21
PM3	3735	348	4.87
NBM1RS	2446	2239	18.87
NBM1RST	2428	3448	24.31
NBM2RS	2487	1501	18.02
NBM2RST	2466	1770	20.71
NBM3RS	2316	605	10.18
NBM3RST	2296	560	10.37

Table A3. DIC, MSE and MAOSPE for the simulation analysis for the window size 3 when time 17 to 19 (Increasing trend)

	**DIC**	**MSE**	**MAOSPE**
PM1	832	67	15.67
PM2	779	53	14.84
PM3	756	45	14.83
NBM1RS	730	63	15.74
NBM1RST	728	61	15.41
NBM2RS	738	68	16.03
NBM2RST	706	57	15.72
NBM3RS	697	53	14.94
NBM3RST	703	55	14.95

Table A4. DIC, MSE and MAOSPE for the simulation analysis for the window size 3 when time 23 to 25 (Decreasing trend)

	**DIC**	**MSE**	**MAOSPE**
PM1	1603	895	26.88
PM2	1212	271	13.34
PM3	1098	175	14.90
NBM1RS	1017	1843	32.31
NBM1RST	1013	1501	32.84
NBM2RS	1040	949	31.11
NBM2RST	1033	922	30.16
NBM3RS	1016	916	30.65
NBM3RST	1020	980	30.29

Table A5. DIC, MSE and MAOSPE table for the analysis of NJ and SC for the window size of 7 (t=33-39 weeks (NJ) and t=11-17 weeks (SC) with increasing trend)

		NJ				SC		
		DIC	MSE	MAOSPE		DIC	MSE	MAOSPE
PM3		1336.62	452.35	32.17		1712.06	63.22	10.61
NBM3RS		1252.77	604.53	123.68		1692.61	73.51	27.82
NBM3RST		1236.55	490.09	124.29		1739.14	64.12	23.25

Table A6. DIC, MSE and MAOSPE table for the analysis of NJ and SC for the window size of 7 (t=9-15 weeks (NJ) and t=20-26 weeks (SC) with decreasing trend)

		NJ				SC		
		DIC	MSE	MAOSPE		DIC	MSE	MAOSPE
PM3		1100.81	92.16	6.28		1892.16	49.11	6.29
NBM3RS		1036.16	102.00	12.25		1872.49	56.31	15.45
NBM3RST		1041.60	103.95	15.84		1852.44	54.94	16.52

Table A7. DIC, MSE and MAOSPE table for the analysis of NJ and SC for the window size of 3 (t=33-35 weeks (NJ) and t=13-15 weeks (SC) with increasing trend)

		NJ				SC		
		DIC	MSE	MAOSPE		DIC	MSE	MAOSPE
PM3		491.28	157.99	25.60		724.61	19.39	8.37
NBM3RS		503.38	197.88	119.86		652.14	18.57	35.85
NBM3RST		512.48	196.36	131.29		654.36	19.57	35.85

Table A8. DIC, MSE and MAOSPE table for the analysis of NJ and SC for the window size of 3 (t=9-11 weeks (NJ) and t=22-24 weeks (SC) with decreasing trend)

		NJ				SC		
		DIC	MSE	MAOSPE		DIC	MSE	MAOSPE
PM3		458.11	84.58	12.83		765.38	23.22	7.20
NBM3RS		481.80	97.30	20.94		1032.19	24.59	7.68
NBM3RST		431.38	87.80	34.10		1058.11	24.70	7.63
